# Clinical efficacy of liver resection after downsizing systemic chemotherapy for initially unresectable liver metastases

**DOI:** 10.1186/s12957-016-0807-7

**Published:** 2016-02-25

**Authors:** Junichiro Kawamura, Takefumi Yazawa, Kimiaki Sumida, Yuya Kida, Ryotaro Ogawa, Masaki Tani, Junya Kawasoe, Michihiro Yamamoto, Hideki Harada, Hidekazu Yamamoto, Masazumi Zaima

**Affiliations:** Department of Surgery, Shiga Medical Center for Adults, 5-4-30 Moriyama, Moriyama, Shiga 524-8524 Japan; Department of Surgery, Faculty of Medicine, Kinki University, 377-2 Ohno Higashi, Osaka, Sayama, Osaka 589-8511 Japan

**Keywords:** Conversion therapy, Colorectal cancer, Liver metastases, Systemic chemotherapy

## Abstract

**Background:**

This study sought to clarify the clinical benefits of liver resection after downsizing systemic chemotherapy for initially unresectable colorectal liver metastases (CLM).

**Methods:**

Survival and clinical characteristics of CLM patients who underwent resection between January 2001 and December 2013 were retrospectively assessed. The study cohort of 88 patients with limited liver disease who underwent curative liver resection comprised 34 with initially resectable synchronous disease (synchronous group), 38 with initially resectable metachronous disease (metachronous group), and 16 with initially unresectable converted disease (conversion group).

**Results:**

The median duration of follow-up for the overall study population was 33 (1–98) months. Overall survival (OS) in the conversion group was not significantly different from that in the other groups. However, disease-free survival (DFS) in the conversion group was significantly shorter than that in the synchronous group. The median DFS was 19.1 months in the synchronous group, 16.6 months in the metachronous group, and 15.3 months in the conversion group. Most patients in the conversion group had recurrence shortly after liver resection in the remnant liver with or without metastases at other sites, but many could undergo repeat hepatectomy or resection of the metastases at other sites.

**Conclusions:**

Although the converted patients tended to have recurrence shortly after liver resection, survival could be prolonged by repeat hepatectomy or resection of metastases at other sites. Liver resection after downsizing chemotherapy appears to be efficacious for patients with initially unresectable CLM and may result in long-term outcomes equivalent to those of patients with initially resectable CLM.

## Background

Colorectal cancer (CRC) is the third most common cause of cancer-related mortality and the second most common malignancy in Japan [1]. Among CRC patients, approximately 25 % present with distant metastases at initial diagnosis and almost 50 % will develop metastases during the disease course [2]. Metastasis is prevalent in the liver. It is present in nearly 80 % of stage IV patients and is the sole site of disease in approximately 40 % of these patients [3]. Complete resection of colorectal liver metastasis (CLM) is considered to be the only curative method of treatment. However, only 20 % of CLM patients are able to undergo resection of metastases; the remaining patients undergo systemic chemotherapy, radiotherapy, or hepatic arterial infusion [4].

Chemotherapy for CRC has improved substantially over the last decade with the development of new cytotoxic drugs and molecular targeted agents, including anti-epidermal growth factor receptor (EGFR) antibody and anti-vascular endothelial growth factor (VEGF) antibody. As a result, systemic chemotherapy is now effective and subsequent surgical resection possible in increasing numbers for patients with initially unresectable CLM [5, 6]. The term “conversion therapy” has been proposed to refer to the use of induction chemotherapy in patients with initially unresectable CLM. Adam et al. reported that among patients with initially unresectable CLM who underwent induction chemotherapy, 12.5 % were subsequently able to undergo liver resection, and of these patients, 16 % could achieve a cure [4, 7]. However, no definitive survival advantage has yet been demonstrated for surgical resection after conversion therapy for initially unresectable CLM. Among the reasons for this are that the definitions of unresectable CLM used vary among institutions and surgeons. Additionally, physicians in different specialties have different treatment preferences for CLM: medical oncologists may favor systemic chemotherapy, whereas surgeons may prefer resection of CLM. Even in a single institution, the indications for surgery might not be consistent. It might also be unrealistic to conduct a clinical trial comparing the outcomes of patients undergoing surgical resection with those of patients who continue with systemic chemotherapy after unresectable CLM becomes resectable, because surgical resection is strongly recommended if CLM is resectable. Therefore, in an effort to clarify the clinical benefit of surgical resection after downsizing systemic chemotherapy for patients with unresectable CLM, we retrospectively assessed the survival and clinical characteristics of surgical patients with CLM and compared them between patients with initially resectable CLM and patients with initially unresectable CLM.

## Methods

### Study design

All patients who underwent liver resection for CLM between January 2001 and December 2013 in the Department of Surgery of Shiga Medical Center for Adults were identified from a prospective surgery database. All identified inpatient and outpatient medical records were then retrospectively reviewed. Colon cancers were diagnosed by colonoscopy with pathologic confirmation. All patients underwent abdominopelvic and thoracic contrast-enhanced computed tomography (CT) scan or contrast-enhanced abdominal magnetic resonance imaging (MRI) and optional positron emission tomography (PET) preoperatively or prior to induction chemotherapy. After liver resection, all patients received at least 3 months of follow-up. CT scans and carcinoembryonic antigen (CEA) and cancer antigen 19-9 (CA19-9) serum levels were assessed during follow-up. Survival was calculated from the date of liver surgery to the last date of follow-up. The Ethics Committee of our institution approved this study.

### Definitions

Contraindications to liver resection for CLM at our institute are (1) remnant liver volume <30 % or estimated liver remnant plasma clearance rate of indocyanine green (ICG-K) <0.5 and (2) the need for reconstruction of a major vessel that needs to be preserved for proper liver function but is invaded by the tumor. Synchronous metastases were defined as CLM diagnosed before colorectal resection or at the time of surgery. In accordance with the guidelines of the International Union Against Cancer, R0 resection was defined by the absence of microscopic tumor invasion of the resection margins (tumor-free margin ≥1 mm for all detected lesions) and R1 resection as the presence of such invasion of the resection margins (tumor-free margin 0 mm) [8]. R2 resection was defined by the presence of macroscopic tumor invasion of the resection margins.

Tumor response was assessed at least every 6 cycles (12 weeks) using the same methods as those performed at baseline (CT, MRI, or PET), according to Response Evaluation Criteria In Solid Tumors (RECIST) criteria [9]. Tumors were assessed for resectability by a multidisciplinary team. Resection was performed within 8 weeks of the last treatment cycle.

### Statistical analysis

Qualitative data were reported as the number of patients and were compared with either the Pearson *χ*2 test or Fisher’s exact test, as deemed appropriate. For qualitative data, the Wilcoxon rank-sum test was used when comparing two groups and the Kruskal–Wallis test when comparing three groups. Survival curves were plotted using the Kaplan–Meier method and compared with the log-rank test. Survival was measured from the date of diagnosis of the liver metastasis until death or last follow-up. Parameters that had an impact on survival were identified by univariate analysis, and those with *p* values less than 0.15 were entered into a Cox proportional hazards model to identify the independent prognostic factors for survival. Tests were always two-sided and the level of statistical significance was set at *p* < 0.05. All analysis was performed using JMP 10 software (SAS Institute Inc., Cary, NC).

## Results

### Demographics

**Fig. 1:**
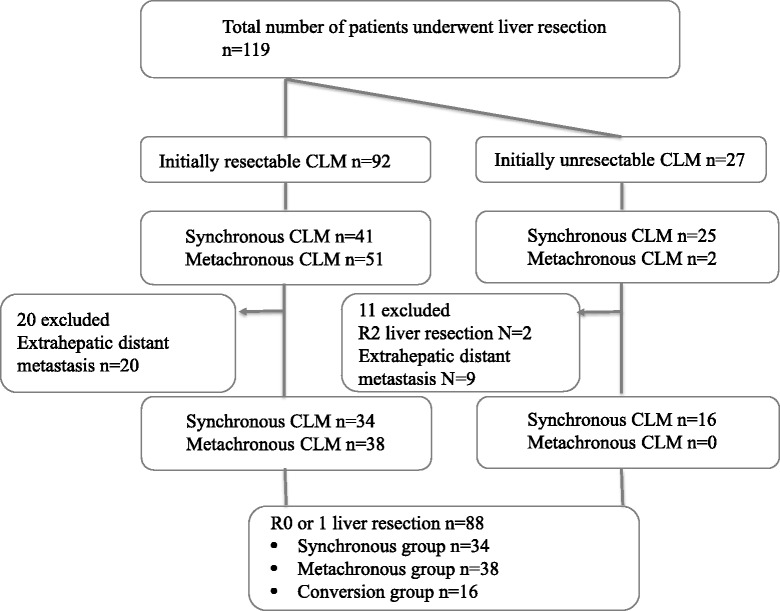
Patient flow diagram. A total of 119 consecutive patients underwent liver resections for CLM in the study period. Thirty-one patients were excluded from the study because of macroscopic residual disease. Of these 31, 7 underwent R2 liver resection and 24 underwent R0 liver resection with concomitant extrahepatic disease at the time of liver resection. Therefore, the study cohort consisted of 88 patients with limited liver disease who underwent curative liver resection: 34 with initially resectable synchronous disease (synchronous group), 38 with initially resectable metachronous disease (metachronous group), and 16 with initially unresectable converted disease (conversion group). *CLM* colorectal liver metastasis

The patient flow diagram is shown in Fig. 1. A total of 119 consecutive patients underwent liver resections for CLM in the study period: 92 had initially resectable CLM and 27 had initially unresectable CLM that had converted after systemic chemotherapy. Thirty-one patients were excluded from the study because of macroscopic residual disease. Although the presence of resectable extrahepatic metastases was not a contraindication for liver resection, patients with macroscopic extrahepatic disease at the time of liver resection were excluded in this study. Of the 31 excluded patients, 29 had extrahepatic distant metastasis at the time of liver resection with or without macroscopic residual disease in the remnant liver and two patients underwent liver resection with macroscopic hepatic residual disease without extrahepatic distant metastasis. Therefore, the study cohort consisted of 88 patients with limited liver disease who underwent curative liver resection: 34 with initially resectable synchronous disease (synchronous group), 38 with initially resectable metachronous disease (metachronous group), and 16 with initially unresectable converted disease (conversion group). All 16 patients in the conversion group had synchronous disease. As shown in Table 1, statistical analysis showed no significant differences between the three groups in terms of sex, *T* number, *N* number, location of primary tumor, tumor size, CEA level, or CA19-9 level. The number of liver metastases and R1 resection rate were significantly different between the three groups (*p* = 0.0001 and *p* = 0.0001, respectively).

**Table 1:** Characteristics of all surgical patients

Variable		Synchronous disease (*n* = 34)	Metachronous disease (*n* = 38)	Conversion disease (*n* = 16)	*p*
Age, years^a^		67.5 (35–85)	63 (35–88)	57.5 (32–80)	0.0458
Sex, *n* (%)					
	Male	22 (64.7 %)	20 (52.6 %)	8 (50 %)	0.487
	Female	12 (35.3 %)	18 (47.4 %)	8 (50 %)	
*T*, *n* (%)					
	T1/2	1 (2.9 %)	3 (7.9 %)	0 (0 %)	0.377
	T3/4	33 (97.1 %)	35 (92.1 %)	16 (100 %)	
*N*, *n* (%)					
	*N*−	13 (38.2 %)	19 (50 %)	7 (43.8 %)	0.603
	*N*+	21 (61.8 %)	19 (50 %)	9 (56.3 %)	
Location of primary tumor, *n* (%)					
	Colon	22 (64.7 %)	23 (60.5 %)	12 (75 %)	0.596
	Rectum	12 (35.3 %)	15 (39.5 %)	4 (25 %)	
Preoperative chemotherapy, *n* (%)					
	Yes	5 (14.7 %)	10 (26.3 %)	16 (100 %)	<0.0001
	No	29 (85.3 %)	28 (73.7 %)	0 (0 %)	
Adjuvant chemotherapy, *n* (%)					
	Yes	16 (47.1 %)	7 (18.4 %)	7 (43.8 %)	0.0252
	No	18 (52.9 %)	31 (81.6 %)	9 (56.3 %)	
No. of liver metastases, *n* (%)					
	1	10 (29.4 %)	22 (57.9 %)	1 (6.3 %)	<0.0001
	2–4	15 (44.1 %)	10 (26.3 %)	2 (12.5 %)	
	≥5	9 (26.5 %)	6 (15.8 %)	13 (81.3 %)	
Size, *n* (%)					
	0–5 cm	28 (82.4 %)	35 (92.1 %)	11 (68.8 %)	0.0946
	>5 cm	6 (17.6 %)	3 (7.9 %)	5 (31.3 %)	
Resection, *n* (%)					
	0	31 (81.6 %)	38 (100 %)	9 (56.3 %)	<0.0001
	1	3 (18.4 %)	0 (0 %)	7 (43.8 %)	
CEA (ng/mL)^a^		17.9 (2.1–2300)	12.45 (1.6–196.3)	11.35 (2.4–7510)	0.729
CA19-9 (U/mL)^a^		19.45 (2–7676)	19.8 (2–7191)	27.3 (2–1063)	0.78

^a^Median (range)

### Survival and recurrence

**Fig. 2:**
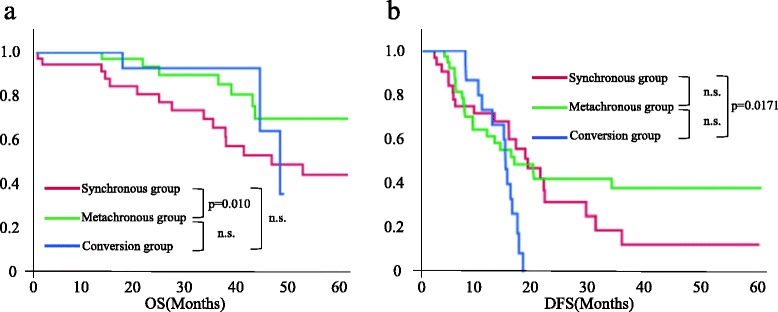
Kaplan–Meier analysis of OS (**a**) and DFS (**b**) in the synchronous, metachronous, and conversion groups. OS in the conversion group was not significantly different from that in the other groups. OS was significantly higher in the metachronous group than that in the metachronous group (*p* = 0.010; Fig. 2a). DFS in the conversion group was significantly shorter than that in the synchronous group (*p* = 0.0171). Median DFS was 19.1, 16.6, and 15.0 months in the synchronous, metachronous, and conversion groups, respectively. *OS* overall survival, *DFS* disease-free survival, *n.s.* not significant

The median duration of follow-up for the overall study population was 33 (1–98) months. Overall survival (OS) in the conversion group was not significantly different from that in the other groups. OS was significantly higher in the metachronous group than that in the synchronous group (*p* = 0.010; Fig. 2a). The median OS was 41 months in the synchronous group and 48.2 months in the conversion group, and the patients in the metachronous group did not reach the median survival. However, disease-free survival (DFS) in the conversion group was significantly shorter than that in the synchronous group (*p* = 0.0171). The median DFS was 19.1 months in the synchronous group, 16.6 months in the metachronous group, and 15.0 months in the conversion group (Fig. 2b). Most patients in the conversion group had recurrence within a year (12/16, 75 %), compared with 28.1 % in the synchronous group and 38.3 % in the metachronous group.

Univariate analysis of all patients demonstrated the significant effect of age >65 years (*p* < 0.001; HR 5.12, 95 % CI 2.34–11.9) and synchronous disease that included cases in both the synchronous and conversion group (*p* = 0.021; HR 2.45, 95 % CI 1.14–5.85) on OS. R1 resection showed a nonsignificant trend (Table 2). A significant factor that negatively influenced DFS was the number of liver metastases ≥5 (*p* = 0.013; HR 2.08, 95 % CI 1.17–3.64), maximum size of metastasis of >5 cm, and conversion disease. Multivariate analysis revealed a significant negative influence on OS of age >65 years (*p* < 0.001; HR 5.90, 95 % CI 2.57–14.5) and synchronous disease (*p* = 0.009; HR 2.85, 95 % CI 1.28–7.03) (Table 3). The number of liver metastases ≥5 showed a nonsignificant trend towards shorter DFS (*p* = 0.137; HR 1.68, 95 % CI 0.85–3.26).

**Table 2:** Univariate analyses of factors associated with OS and DFS in all surgical patients

	OS			DFS		
	HR	95 % CI	*p*	HR	95 % CI	*p*
Age >65 years	5.12	2.34–11.9	<0.001	1.37	0.80–2.33	0.241
Male	1.87	0.89–4.31	0.100	1.18	0.70–2.04	0.538
Synchronous disease	2.45	1.14–5.85	0.021	1.50	0.87–2.65	0.141
Conversion disease	1.56	0.36–4.75	0.504	1.99	1.02–3.70	0.0435
Node-positive primary tumor	1.43	0.67–2.99	0.342	1.30	0.77–2.26	0.333
Rectum	0.83	0.37–1.74	0.897	1.11	0.71–1.92	0.709
Adjuvant chemotherapy	0.78	0.36–1.61	0.506	0.90	0.50–1.53	0.693
No. of liver metastases ≥5	1.43	0.63–3.06	0.164	2.18	1.23–3.79	0.0076
Maximum tumor size >5 cm	1.32	0.48–3.15	0.555	1.99	1.04–3.56	0.0375
CA19-9 ≥100 U/mL	1.40	0.55–3.13	0.457	1.46	0.77–2.63	0.235
R1 resection	1.07	0.51–2.17	0.853	1.15	0.68–1.96	0.597

*OS* overall survival, *DFS* disease-free survival, *HR* hazard ratio, *CI* confidence interval

**Table 3:** Multivariate analysis of factors associated with OS and DFS in all surgical patients

OS	HR	95 % CI	*p*
Age >65 years	5.90	2.57–14.5	<0.001
Male	1.12	0.52–2.62	0.780
Synchronous disease	2.85	1.28–7.03	0.009
DFS	HR	95 % CI	*p*
Synchronous disease	1.08	0.57–2.05	0.809
Conversion disease	1.32	0.59–2.90	0.491
No. of liver metastases ≥5	1.68	0.85–3.26	0.137
Maximum tumor size >5 cm	1.59	0.80–3.02	0.179

*OS* overall survival, *DFS* disease-free survival, *HR* hazard ratio, *CI* confidence interval

### Conversion group characteristics

**Fig. 3:**
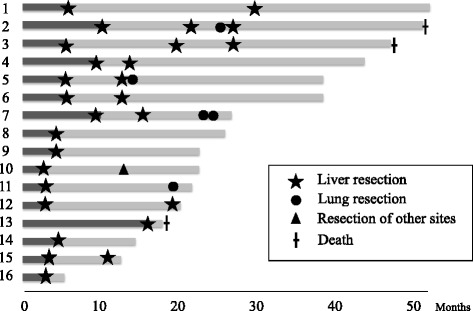
Clinical course of patients in the conversion group after liver resection. The majority of these patients had recurrence shortly after liver resection in the remnant liver with or without metastases at other sites, but most of them could undergo repeat hepatectomy or resection of metastases at other sites

Patient characteristics in the conversion group are shown in Table 4. All 16 patients had synchronous disease, 13 of whom had ≥5 tumors in the liver. Also, patients in the conversion group had a larger maximum tumor size than those in the other groups. The regimens that were initially used for the converted patients were FOLFOX6 plus cetuximab in seven patients, FOLFIRI plus cetuximab in three patients, FOLFOX6 plus bevacizumab in five patients, and FOLFOX6 plus panitumumab in one patient. The median number of cycles was 3.5 (2–18 cycles). All except one patient underwent just one line of chemotherapy before their unresectable CLM became resectable. The clinical course after liver resection is shown in Fig. 3. Most patients in the conversion group, aside from those with a short follow-up period, had recurrence shortly after liver resection in the remnant liver with or without metastases at other sites, but many of them could undergo repeat hepatectomy or resection of the metastases at other sites.

**Table 4:** Patients characteristics in the conversion group

	Age (years)	Sex	Primary	T category	Node positive	Tumor size (mm)	Tumor number	Regimen	No. of lines	No. of cycles	Clinical response	Resection
1	65	M	Colon	3	+	80	≥5	Cmab + FOLFIRI	1	3	PR	1
2	57	M	Rectum	3	+	50	≥5	Bmab + FOLFOX6	1	17	PR	0
3	52	F	Colon	4	+	30	≥5	Cmab + FOLFIRI	1	3	PR	1
4	51	M	Colon	3	−	20	2–4	Bmab + FOLFOX6	1	18	PR	0
5	60	M	Rectum	4	+	50	≥5	Cmab + FOLFOX6	1	7	PR	1
6	67	M	Colon	4	−	30	≥5	Cmab + FOLFOX6	1	8	PR	1
7	78	M	Colon	3	−	20	≥5	Cmab + FOLFOX6	1	15	PR	1
8	32	F	Colon	3	+	100	≥5	Bmab + FOLFOX6	1	8	SD	1
9	80	F	Colon	3	−	32	≥5	Bmab + FOLFOX6	1	4	PR	0
10	44	F	Colon	3	+	100	≥5	Cmab + FOLFOX6	1	3	SD	0
11	33	F	Colon	3	+	24	≥5	Bmab + FOLFOX6	1	2	PR	0
12	56	F	Colon	3	−	80	2–4	Cmab + FOLFOX6	1	3	PR	0
13	58	M	Colon	3	+	40	≥5	Cmab + FOLFIRI/Bmab + Xelox	2	18	PR	1
14	55	F	Colon	4	−	55	≥5	Cmab + FOLFOX6	1	3	PR	0
15	58	F	Rectum	3	+	40	1	Cmab + FOLFOX6	1	3	SD	0
16	65	M	Rectum	3	−	23	≥5	Pmab + FOLFOX6	1	3	PR	0

*M* male, *F* female, *Cmab* cetuximab, *Bmab* bevacizumab, *PR* partial response, *SD* stable disease

## Discussion

This comparative study of the outcomes of liver resection for initially resectable and unresectable CLM found that OS was comparable between patients who underwent liver resection for initially resectable CLM and those who underwent liver resection after downsizing systemic chemotherapy for initially unresectable CLM. The converted patients tended to have recurrence shortly after liver resection; however, their survival might be improved through repeat hepatectomy or resection of metastases at other sites. These results support the clinical efficacy of liver resection after downsizing chemotherapy for patients with unresectable CLM.

The criteria for resectability not only differed between previous studies but also were often poorly defined [10]. Although general principles were applied to the criteria for resectability in our institution, the decision regarding resectability could be made based on personal judgment in the clinical setting. Not surprisingly, judgment of conversion from unresectable to resectable status is somewhat subjective. Therefore, since palliative hepatectomy without curative intent might have been provided for some patients with aggressive symptomatic liver metastases in addition to uncontrolled macroscopic extrahepatic disease, patients with macroscopic extrahepatic disease at the time of liver resection and those with R2 liver resection were excluded to enable comparison of patients who underwent treatment with curative intent alone.

Although no prospective randomized controlled trials have demonstrated the efficacy of conversion therapy, some surgical series have reported favorable results of liver resection after downsizing chemotherapy for initially unresectable CLM. A systematic review reported a median OS of 45 months (range, 36–60 months) and DFS of 14 months, although the majority of the reviewed studies were published a decade ago [10]. Recent studies have reported a median OS of 40.5–57.6 months and DFS of 3.2–10.6 months, indicating significantly shorter median DFS in the conversion group than in the initially resectable group but a comparable median OS [11, 12]. The present study reaffirms the finding of prolonged survival of patients in the conversion group despite their relatively shorter DFS, which we attribute to repeat hepatectomy or resection of metastases at other sites.

Multivariate analysis identified two significant factors of OS (age >65 years and synchronous disease). A number of previous reports have shown that several prognostic factors affect patient outcome, and similar factors were noted in this study [13–18]. The presence of multiple hepatic metastases has been reported to be a very important risk factor for recurrence after liver resection, and the results in this study showed a similar trend but did not reach statistical significance [19, 20]. In the present study, R1 resection was not a significant risk factor that affected both OS and DFS. Although many publications have reported microscopic involvement of surgical resection margins as a significant poor prognostic factor, whether R1 resection is contraindicated remains a controversial issue [13, 20–25]. Some authors have justified intended R1 resection combined with increasingly efficient chemotherapy regimens [21, 26, 27]. Our study also suggests that R1 resection is not a contraindication for resection and that resection of all liver metastasis in converted patients may be effective even if the resection margin becomes microscopically positive.

In previous studies, the addition of molecular targeted drugs, anti-epidermal growth factor receptor (EGFR) agents such as cetuximab or panitumumab, or the anti-vascular endothelial growth factor (VEGF) agent bevacizumab to doublet chemotherapy such as FOLFOX or FOLFIRI achieved a higher response than doublet chemotherapy alone, resulting in a higher R0 resection rate for patients with initially unresectable CLM [5, 6]. In our institution, KRAS wild-type patients who had unresectable CLM alone or unresectable CLM with controllable extrahepatic metastases were to receive FOLFOX6 plus anti-EGFR agents in principle in the second half of the study period. All of the converted patients received doublet chemotherapy in combination with molecular-targeted drugs as first-line chemotherapy. Of these 16 patients, 11 had KRAS wild-type tumors and received FOLFOX6 or FOLFIRI plus anti-EGFR agents and 5 had KRAS mutant-type tumors and received FOLFOX6 plus bevacizumab. Although limited findings are available so far on the potency of these new regimes as conversion therapy, modern systemic chemotherapy has definitely increased conversion rates. Future studies should address which regimens are optimal for achieving conversion of patients with initially unresectable CLM.

This study is not without limitations. First, the conversion group was small. To determine the clinical efficacy of liver resection after downsizing chemotherapy, we must accumulate data from more cases to achieve adequate statistical power. Second, this study was not a comparison of outcomes between surgical patients and patients who continued chemotherapy after unresectable CLM was rendered resectable. In assessing the value of resection after “conversion therapy,” appropriate control patients might be converted patients who continued chemotherapy without liver resection. However, it is unlikely that a prospective randomized trial comparing these outcomes will be conducted since surgical resection is strongly recommended if CLM is deemed resectable. Third, this was a retrospective study and selection bias is possible. The patients in the conversion group were younger and might have been selected because of their good general condition aside from their cancer. Fourth, the chemotherapy regimen used in the study period has changed and the impact of modern chemotherapy on survival could not be determined. Finally, we did not collect data from patients who had unresectable CLM with or without extrahepatic metastases, so the true number of patients with unresectable disease and the conversion rate are unclear.

## Conclusions

Although the converted patients tended to have recurrence shortly after liver resection, their survival could be prolonged by repeat hepatectomy or resection of metastases at other sites. Liver resection after downsizing chemotherapy for patients with unresectable CLM appears to be efficacious and may result in long-term outcomes equivalent to those of patients with initially resectable CLM.
